# New records of fifteen species of Fulgoromorpha (Insecta: Hemiptera) in Bulgaria

**DOI:** 10.3897/BDJ.10.e83231

**Published:** 2022-05-09

**Authors:** Ilia Gjonov

**Affiliations:** 1 Sofia University, Faculty of Biology, Sofia, Bulgaria Sofia University, Faculty of Biology Sofia Bulgaria

**Keywords:** Fulgoroidea, fauna, the Balkans, Cixiidae, Delphacidae, Issidae, Dictyopharidae

## Abstract

**Background:**

Bulgarian planthopper fauna (Hemiptera: Fulgoromorpha) are relatively well studied, with 164 known species from 77 genera and 11 families. Data for some species from previous studies were reported without any localities or were incomplete and need to be updated.

**New information:**

In the present study, 13 species of planthoppers are recorded for the first time in Bulgaria - *Hyalesthesmlokosiewiczi* Signoret, 1879 (Cixiidae), *Delphaxarmeniacus* Anufriev, 1970, *Euidesspeciosa* (Boheman, 1845), *Eurysulalurida* (Fieber, 1866), *Florodelphaxparyphasma* (Flor, 1861), *Jassidaeuslugubris* (Signoret, 1865), *Metropisaris* Asche, Drosopoulos & Hoch, 1983, *Oncodelphaxpullula* (Boheman, 1852), *Ribautodelphaximitans* (Ribaut, 1953), *R.pungens* (Ribaut, 1953), *Stenocranusmajor* (Kirschbaum, 1868) (Delphacidae), *Latilicamaculipes* (Melichar, 1906) and *Tshurtshurnellaextrema* Dlabola, 1980 (Issidae). Species from the following five genera are recorded in Bulgaria for the first time: *Euides* Fieber, 1866, *Eurysula* Vilbaste, 1968, *Jassidaeus* Fieber, 1866, *Oncodelphax* Wagner, 1963 (Delphacidae) and *Latilica* Emeljanov, 1971 (Issidae). As a result, the total numbers of known planthopper species and genera in Bulgaria become 177 species and 82 genera. The dataset of all collected specimens presented in this work was provided separately through Global Biodiversity Information Facility (GBIF). Detailed distribution of the species and comments on those from the European Red Lists are also provided.

## Introduction

Fulgoromorpha (planthoppers) are hemimetabolous insects belonging to the order Hemiptera. They are widespread throughout the world, but most families are richer in the tropics. About 14,000 species of fulgoromorphs have been described worldwide, belonging to 36 families (including fossils) (Bourgoin 2022) and about 730 species from 13 families are known in Europe (Hoch 2013). In Bulgaria, as well as on the Balkan Peninsula as a whole, insects of the infraorder Fulgoromorpha have been insufficiently studied from faunal, taxonomic and biological points of view. Data from older studies are incomplete and often need to be confirmed. There are no faunal lists, monographs, identification keys or other overview publications for Bulgaria. This, as well as the economic importance of the group, necessitates a comprehensive and up-to-date study of the Fulgoromorpha species in Bulgaria.

## Materials and methods

The material was collected in Bulgaria between 2003 and 2021 by using sweeping nets and light towers. After collection, they were preserved in dry conditions on cotton mattresses. The specimens were dry-mounted on paper boards after humidification. Dissections were performed when necessary for identification and the dissected genitalia were glued to the boards. Due to the lack of identification keys for Balkan Fulgoromorpha, identification data were used from various publications (Holzinger et al. 2003, Biedermann and Niedringhaus 2009, Emeljanov 2015). The specimens are digitised and stored at the Zoological Collection of Sofia University (BFUS). The dataset of all records presented in this work has been published separately through Global Biodiversity Information Facility (GBIF) (Gjonov 2022). Each entry of the dataset includes a single collection specimen with individual collection number, geospacial information, date of collecting, storing collection and taxonomic affiliation.

Detailed distribution of the species and comments on those from the European Red Lists are also provided. The following abbreviations concerning conservation status of the species where used: **CR** - Critically Endangered (IUCN) and category "2" in German Red Lists, **EN** - Endangered (IUCN) and category "2" in German Red Lists, **VU** - Vulnerable and category "V" in German Red Lists.

Some species were photographed live by the author with a Canon EOS 70D DSLR camera, Canon MP-E 65 mm macro lens using an Yongnuo YN-24EX twin macro flash or with Olympus E-500 DSLR camera, Sigma 150mm F2.8 APO MACRO DG and Raynox DCR-250 macro lens attached using Bower SFD14C ring flash.

## Checklists

### Fulgoromorpha Evans, 1946

#### Cixiidae

Spinola, 1839

284F3298-CDB3-50F9-A9ED-7437682E5AE0

#### Hyalesthes

Signoret, 1865

18FD2692-8E74-5F63-AC6F-C72C6F70414C

#### Hyalesthes mlokosiewiczi

Signoret, 1879

424E02B9-2A84-52EA-834D-5ABBC0EA5566

##### Distribution

Greece (Rhodos), Georgia (Melichar 1914), Cyprus, Lebanon, Iraq, Israel as *H.mavromoustakisi* and *H.aither* (Dlabola 1959, Hoch 1990), Turkey (Lodos and Kalkandelen 1980, Demir 2007, Demir and Demirsoy 2009, Demirel and Hasbenli 2015), Iran (Dlabola 1981, Mozaffarian and Wilson 2011, Mozaffarian 2018), Turkmenistan, Uzbekistan, Kyrgyzstan (Dlabola 1958), Armenia (Lindberg 1960), Azerbaijan, Russia (Krasnodar, Dagestan) (Emeljanov 2015).

##### Notes

First record for Bulgaria. **Western Danube Plain**: Guljantsi vill., 07.viii.2021, 2♂♂ and 2♀♀ (Fig. 1); **Western Rhodopes Mts.**:, Djadovci vill., 26.v.2014, 9♂♂ and 4♀♀; Bachkovo vill., 29.v.2021, 1♂; **Eastern Rhodopes Mts.**: Zimzelen vill., 25.v.2014, 3♂♂ and 1♀; Bjal Gradec vill., 02.vi.2015, 2♂♂ and 1 nymph; Dolno Lukovo vill., 01.vi.2015, 1♂. All specimens were collected on *Salix* shrubs. Detailed occurrence data: Gjonov (2022).

This species is recorded as a pest in the Caucasus (Emeljanov 2015) and Iran (Mozaffarian 2014, Mozaffarian 2018). Although it is hardly a danger to crops in Bulgaria (being found only on willow bushes), it should be monitored in the future.

**Red Lists**: No assessment.

**Host plant**: Polyphagous (Emeljanov 2015).

#### Delphacidae

Leach, 1815

F0828B3C-4E7F-5D9C-89F3-6BDE6C1E3725

#### Delphax

Fabricius, 1789

FA78AB25-5F05-52DF-886F-06B942C0E86C

#### Delphax armeniacus

Anufriev, 1970

4AF027A0-5AB7-51D0-AE7F-1C81D475F4FD

##### Distribution

Ukraine (Nast 1987), Greece (Drosopoulos 1982b, Drosopoulos et al. 1983), Armenia (Anufriev 1970), North-western Caucasus, Kazakhstan (Mitjaev 2015).

##### Notes

First record for Bulgaria. **Southern Black Sea coast**: Sinemorec vill., the mouth of Veleka River, at light, near a marsh, 14.viii.2010, 1♂. Detailed occurrence data: Gjonov (2022).

**Red Lists**: No assessment.

**Host plant**: There are no literature data. Although the specimen was collected in light, it is assumed that it came from the nearest *Phragmitesaustralis* (Cav.) Trin. ex Steud. plantation, the typical host-plant for *Delphax* species (Nickel 2003b).

#### Euides

Fieber, 1866

18721F12-F1A9-5D13-9598-6E7B9F308E3B

#### Euides speciosa

(Boheman, 1845)

35420FBA-CC70-56DE-AA67-AE6D02BF4748

Della Giustina (2019) does not support the synonymy of *E.basilinea* (Germar, 1821) under *E.speciosa* proposed by Nast (1986).

##### Distribution

Norway, Denmark, Sweden, Finland, Estonia, Latvia, Lithuania, Russia (Karelia) (Söderman et al. 2009), Germany, Austria, Switzerland (Mühlethaler et al. 2018), Luxembourg (Niedringhaus et al. 2010a), as *E.basilinea* (Germar, 1821). Synonimisation is refuted by Della Giustina (2019) for the North of Europe. France (Della Giustina 2019), Hungary (Asche 1982a), ex-Yugoslavia (Asche 1982b), Belarus (Borodin 2004), Kazakhstan (Mitjaev 1971), Korea (Park and Jung 2020), Japan (Hayashi and Fujinuma 2016).

##### Notes

New record for Bulgaria. **Western Danube Plain**: Archar vill., 04.v.2015, 1♂; **Southern Black Sea coast**: Atia vill., 22.viii.2016, 1♂. Detailed occurrence data: Gjonov (2022).

**Red Lists: EN**: Saxony (Walter et al. 2003); **VU**: (under the name *E.basilinea*): Bavaria (Nickel 2003a), Tyrphobionts and Tyrphophils of Hanoverian Moor Geest (Nickel and Gärtner 2009), Watercourses and Springs on the Hoher Trauchberg, Eastern Allgäu/Bavarian Alps (Bückle and Guglielmino 2011) and Germany (Nickel et al. 2016).

**Host plant**: *Phragmitesaustralis* (Della Giustina 2019).

#### Eurysula

Vilbaste, 1968

279E9B72-DF85-5D7B-826D-71661BD81619

#### Eurysula lurida

(Fieber, 1866)

6D319526-D699-5381-B087-FA2F506D6B0F

##### Distribution

Norway, Denmark, Sweden, Finland, Estonia, Lithuania, Latvia, Russia (Karelia) (Söderman et al. 2009), Great Britain (Le Quesne and Payne 1981), Ireland, Belgium, France (Della Giustina 2019), Netherlands (Gravestein 1976), Luxembourg (Niedringhaus et al. 2010a, Niedringhaus et al. 2010b), Germany (Nickel and Remane 2002), Poland (Gębicki et al. 2013, Walczak 2016), Ukraine (Logvinenko 1975), Spain (Aguin-Pombo et al. 2007, Aguin-Pombo et al. 2008, Italy (Sicily) (D'Urso 1995), Switzerland (Mühlethaler et al. 2018), Austria (Holzinger 1996a, Holzinger 1996b, Kahapka and Kunz 2011, Kunz and Kahapka 2012, Holzinger et al. 2017), Czech Republic (Malenovský 2006, Malenovský et al. 2011, Malenovský and Lauterer 2012), Hungary (Asche 1982a, Orosz 2008, Orosz 2009), Slovenia (Seljak 2016), Kazakhstan, Mongolia (Mitjaev 2015), ex-Yugoslavia (Asche 1982b).

##### Notes

First record for Bulgaria. **Eastern Sub-Balkan Basins**: Ajtos, 23.vi.2016, 11♂♂, 1♀ and 4 nymphs; **Western Rhodopes Mts**: Poljana vill., 28.v.2014, 2♂♂, 2♀♀ and 5 nymphs. Detailed occurrence data: Gjonov (2022).

**Red Lists**: It is assessed as not endangered in some countries of Central Europe.

**Host plant**: *Calamagrostisepigeios* (L.) Roth, *C.canescens* (Weber ex F.H. Wigg.) Roth (Nickel 2003b).

#### Florodelphax

Vilbaste, 1968

E20AE414-87C6-514B-BD4F-F2DD00DB3100

#### Florodelphax paryphasma

(Flor, 1861)

A0E055F8-1160-5AF6-9A2D-2E781A5870A4

##### Distribution

Sweden, Finland, Estonia, Lithuania, Latvia, Russia (Karelia) (Söderman et al. 2009), Luxembourg (Niedringhaus et al. 2010a), Belgium (Baugnée 2004), France (Della Giustina and Remane 2001), Austria (Holzinger and Kunz 2006), Czech Republic (Preisler and Lauterer 2003, Malenovský and Lauterer 2010), Slovenia (Holzinger and Seljak 2001), ex-Yugoslavia (Asche 1982b), Kazakhstan, Kyrgyzstan, Baikal (Irkutsk) (Mitjaev 2015).

##### Notes

First record for Bulgaria. **Sarnena Sredna Gora**: Svezhen vill., marsh, 11.viii.2020, 2 ♂♂. Detailed occurrence data: Gjonov (2022).

**Red Lists: CR**: Saxony (Walter et al. 2003), Austria (Holzinger 2009); Czech Republic (Malenovský and Lauterer 2017); **EN**: Bavaria (Nickel 2003a), Saxony-Anhalt (Witsack and Nickel 2004), Thuringia (Nickel and Sander 2016).

**Host plant**: On *Carexdisticha* Huds. (Nickel 2003b).

#### Jassidaeus

Fieber, 1866

57F2BB61-5572-5B80-8A43-5673459264AB

#### Jassidaeus lugubris

(Signoret, 1865)

97D9BB1A-B99D-53BC-876E-094759D3E170

##### Distribution

Belgium, France (Della Giustina 2019), Luxembourg (Niedringhaus et al. 2010a), Germany (Nickel and Remane 2003), Poland (Gębicki et al. 2013), Ukraine (Logvinenko 1975), Russia (European parts) (Emel'yanov 1967, Smirnova and Anufriev 2014), Spain, Portugal (Remane and Fröhlich 1994), Italy (Sicily) (D'Urso 1995), Austria (Holzinger 1996b, Holzinger and Kunz 2006), Czech Republic, Slovakia (Dlabola 1977, Malenovský and Lauterer 2012), Hungary (Asche 1982a, Orosz 2009), Romania (Orosz and Tóth 2016), Greece (Drosopoulos et al. 1983).

In the General Catalogue of the Hemiptera (Metcalf 1943) is probably mistakenly recorded for Ceylon without referring to the literature source.

##### Notes

First record for Bulgaria. **Western Pre-Balkan**: Rumjancevo vill., 01.x.2016, 1♂; **Belasitsa Mt**: Varshilo loc., 01.i.2014, 7♂♂ and 4♀♀ (Fig. 2). Detailed occurrence data: Gjonov (2022).

**Red Lists: CR**: Saxony (Walter et al. 2003), Austria (Holzinger 2009), Turingia (Nickel and Sander 2016), Germany (Nickel et al. 2016); **EN**: Bavaria (Nickel 2003a), Saxony-Anhalt (Witsack and Nickel 2004); **VU**: Czech Republic (Malenovský and Lauterer 2017).

**Host plant**: *Festucaovina* L. and perhaps also *Stipacapillata* L. (Nickel 2003b).

#### Metropis

Fieber, 1866

8430A70D-730A-5961-92CD-CCB23219859E

#### Metropis aris

Asche, Drosopoulos & Hoch, 1983

59AB60F6-37D7-5A2A-9A19-CC3AE7B41585

##### Distribution

Greece (Drosopoulos et al. 1983), Slovenia (Seljak 2004, Seljak 2016)

##### Notes

First record for Bulgaria. **Strandzha Mt**: Goljamo Bukovo vill., 05.v.2009, 1♂. Detailed occurrence data: Gjonov (2022).

**Red Lists**: No assessment.

**Host plant**: Unknown.

#### Oncodelphax

Wagner, 1963

1FD7C375-D754-5232-9243-261A35E77144

#### Oncodelphax pullula

(Boheman, 1852)

446EE6AE-67CA-545E-A80D-A031AD0E0F08

##### Distribution

Norway, Denmark, Sweden, Finland, Estonia, Lithuania, Latvia, Russia (Karelia and Leningrad Region) (Söderman et al. 2009), Poland (Gębicki et al. 2013),Germany (Nickel and Remane 2003), Great Britain, Ireland, France, Belgium, Switzerland (Della Giustina 2019), Belarus (Borodin 2004), Austria (Kunz and Plank 2002), Czech Republic (Malenovský et al. 2014), Slovenia (Seljak 2016), Hungary (Asche 1982a), Romania (Orosz and Tóth 2016).

##### Notes

**Figure 3.**
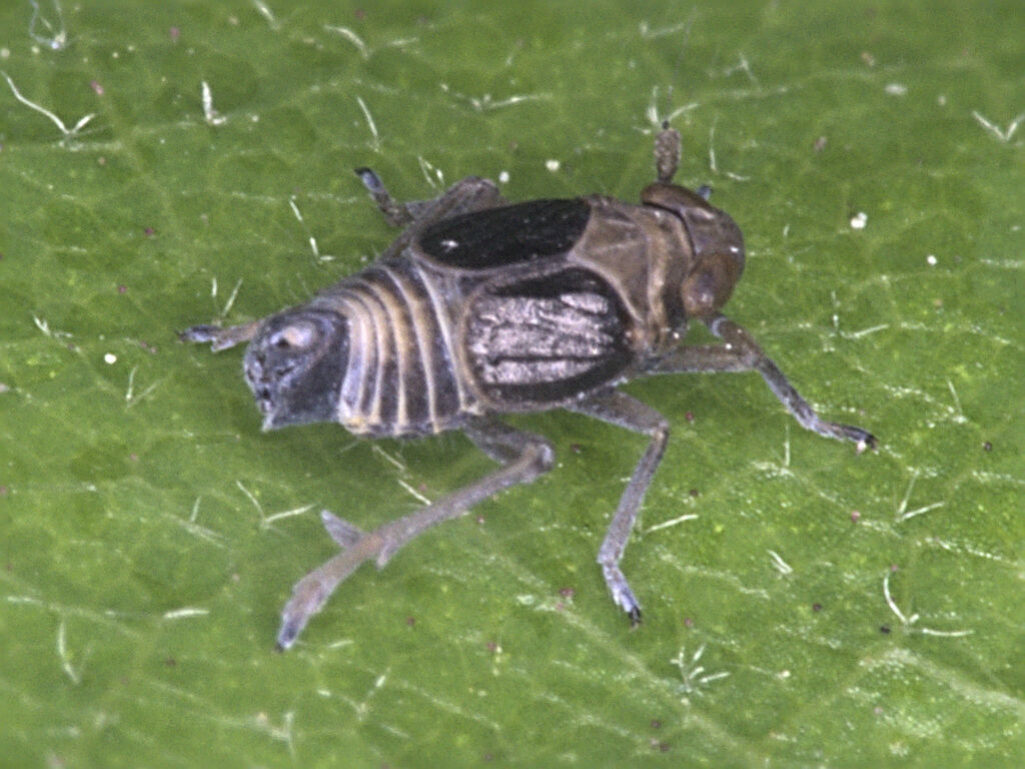
*Oncodelphaxpullula* (Boheman, 1852), male - Bulgaria, Strandzha Mt.

First record for Bulgaria. **Strandzha Mt**: Goljamo Bukovo vill., 05.v.2009, 10♂♂ and 7♀ (Fig. 3). Detailed occurrence data: Gjonov (2022).

**Red Lists: EN**: Carinthia (Austria) (Holzinger 1999), Bavaria (Nickel 2003a), Saxony (Walter et al. 2003), Saxony-Anhalt (Witsack and Nickel 2004), Austria (Holzinger 2009), Germany (Nickel et al. 2016), Watercourses and Springs on the Hoher Trauchberg, Eastern Allgäu/Bavarian Alps (Bückle and Guglielmino 2011); **VU**: Czech Republic’s Red List (Malenovský and Lauterer 2017).

**Host plant**: Mainly *Carexnigra* (L.) Reichard (Nickel 2003b).

#### Ribautodelphax

Wagner, 1963

B67E28EA-9BF5-5C68-901D-C4E33688B566

#### Ribautodelphax imitans

(Ribaut, 1953)

B9C5C09B-969D-5614-BFC2-ED4ADA1269B9

##### Distribution

Great Britain (Le Quesne and Payne 1981), Belgium (Della Giustina 2019), Netherlands (den Bieman and Mol 2010), Luxembourg (Niedringhaus et al. 2010a), Switzerland (Mühlethaler et al. 2018), Germany (Nickel and Remane 2002), Poland (Gębicki et al. 2013), Spain (Aguin-Pombo et al. 2007), France (den Bieman 1987), Italy (Guglielmino et al. 2005, Guglielmino and Bückle 2008, Carl 2008), Austria (Holzinger 1996b, Holzinger et al. 2020), Czech Republic (Malenovský and Lauterer 2010, Malenovský and Lauterer 2012), Hungary (Györffy et al. 2009), Romania (Orosz and Tóth 2016), Slovenia (Holzinger and Seljak 2001), Croatia (Nast 1987), Greece (den Bieman 1987), Kazakhstan (Mitjaev 2015).

##### Notes

First record for Bulgaria. **Sarnena Sredna Gora Mt**: Prjaporets vill., 14.viii.2020, 1♂. Detailed occurrence data: Gjonov (2022).

**Red Lists: EN**: Bavaria (Nickel 2003a); **VU**: Austria (Holzinger 2009).

**Host plant**: FestucaarundinaceaSchreb.subsp.fenas (Lag.) Arcang. (den Bieman 1987).

#### Ribautodelphax pungens

(Ribaut, 1953)

018D01EA-F481-5FBE-A518-973E6E82E25D

##### Distribution

Sweden (Söderman et al. 2009), Netherlands, Belgium, Germany, France, Slovenia, Croatia, Bosnia and Herzegovina, Serbia (Della Giustina 2019), Luxembourg (Niedringhaus et al. 2010a), Poland (Gębicki et al. 2013), Russia (European parts) (Smirnova and Anufriev 2014), Great Britain (Le Quesne and Payne 1981), Switzerland (Mühlethaler et al. 2018), Spain (Aguin-Pombo et al. 2007), France (Corsica) (Bonfils and Della Giustina 1978), Italy (Guglielmino et al. 2005, Guglielmino and Bückle 2008), Austria (Holzinger 1996b, Kunz and Plank 2002), Czech Republic (Malenovský 2006, Malenovský et al. 2011), Hungary (Orosz 2009), Greece (den Bieman 1988).

##### Notes

First record for Bulgaria. **Sarnena Sredna Gora**: Prjaporets vill., 14.viii.2020, 1♂; **Eastern Rhodopes**: Kokiche vill., 06.v.2003, 1♂; **Strandzha Mt**: Izgrev vill., Marina reka loc., 08.v.2009, 2♂♂ and 2♀♀. Detailed occurrence data: Gjonov (2022).

**Red Lists: EN**: Saxony (Walter et al. 2003).

**Host plant**: different *Brachypodium* species (den Bieman 1987); monophagous on *Brachypodiumpinnatum* (L.) P. Beauv (Nickel 2003b).

#### Stenocranus

Fieber, 1866

7547CDAC-04AA-5A22-90E0-6FA072AB0DD6

#### Stenocranus major

(Kirschbaum, 1868)

B9FB7414-103E-5FB0-B516-AF82E404101E

##### Distribution

Norway, Denmark, Sweden, Finland, Latvia (Söderman et al. 2009), Ireland, Great Britain, Belgium, Switzerland, Ukraine, Spain, France (Della Giustina 2019), Netherlands (den Bieman 1993, den Bieman et al. 2021), Russia (European parts) (Anufriev and Bayanov 2002, Söderman et al. 2009), Belarus (Borodin 2004), Luxembourg (Niedringhaus et al. 2010a), Poland (Gębicki et al. 2013), Czech Republic (Dlabola 1954), Germany (Nickel and Remane 2003), Italy (D'Urso 1995, Guglielmino et al. 2005), Austria (Holzinger 1996b, Kunz 2010), Hungary (Asche 1982a), Romania (Popa and Popa 2002), Slovenia (Holzinger and Seljak 2001), ex-Yugoslavia (Asche 1982b), Serbia (Cvrković et al. 2010, Cvrković et al. 2011), Iran (Mozaffarian and Wilson 2011), Kyrgyzstan (Anufriev 2002), Malaysia (Bartlett 2009).

##### Notes

First record for Bulgaria. **Western Pre-Balkan**: Belgradchishki Skali, 03.v.2015, 1♂; **Western Stara Planina**: Slivnitsa vill., Aldomirovsko Blato, 18.iii.2017, 3♂♂ and 10♀♀; same location, 10.vii.2011, 1♂; **Middle Stara Planina**: Divchovoto vill., 08.v.2015, 1♂ and 1♀. Detailed occurrence data: Gjonov (2022).

**Red Lists**: It is assessed as not endangered in some countries of Central Europe.

**Host plant**: *Phalarisarundinacea* L. (Nickel 2003b).

#### Tropidocephala

Stal, 1853

31F63547-B88F-5DB9-BC8F-2BBA034493CE

#### Tropidocephala andropogonis

Horváth, 1895

C6125B1F-0829-569D-B0D1-31BFB11D941D

##### Distribution

Slovakia (Dlabola 1950), Hungary (Horváth 1895), Czech Republic (Dlabola 1977), ex-Yugoslavia (Nast 1972), Bulgaria (Asche 1982b), Greece (Drosopoulos 1982a, Drosopoulos 1982b), Turkey (Dlabola 1981).

##### Notes

First exact locality data for Bulgaria. **Lozenska Mt**: Dolni Pasarel vill., 16.vi.2014, 2♂; **Vlahina Mt**: above Boboshevo, Jana hut, 11.v.2010, 1♂; **Eastern Rhodopes**: Valkovich vill., 24.v.2014, 4♀♀ (Fig. 4). **Strandzha Mt**: Goljamo Bukovo vill., 05.v.2009, 1♂; Izgrev vill., 09.v.2012, 1♂ and 2♀. Detailed occurrence data: Gjonov (2022).

**Red Lists**: No assessment.

**Host plant**: *Chrysopogongryllus* (L.) Trin., 1820 (Horváth 1895, Drosopoulos et al. 1983), also rcorded on *Bothriochloaischaemum* (L.) Keng (Drosopoulos 1982a).

#### Dictyopharidae

Spinola, 1839

87D7A25E-A5D2-5EDE-B994-E1441AB4CC37

#### Dictyophara

Germar, 1833

CFA6C12A-8A4A-5790-B0C6-0F3E7BD5AFE6

#### Dictyophara pannonica

(Germar, 1830)

3F40565E-1A64-57DA-A792-6C167F76249A

##### Distribution

Italy (doubtful) (D'Urso 1995, Lessio and Alma 2008), Slovakia (Dlabola 1977), Hungary (Guglielmino et al. 2013), Romania (Orosz and Tóth 2016), Bulgaria (Nast 1987), Russia (South European Russia, Western Siberia), Kazakhstan, Kyrgyzstan, Mongolia (Mitjaev 2015), Ukraine (Logvinenko 1975), Georgia (Dlabola 1958), Turkey (Dlabola 1957), NW China (Song and Liang 2008).

##### Notes

First exact locality data for Bulgaria. **Kozhuh Hill**: Rupite vill., 11.ix.2021, 2♀♀ (Fig. 5); **Western Rhodopes**: Novo selo vill., Besaparski Hills, 24.vii.2010, 2♂; same place, 07.vii.2012, 1 nymph; same place, 14.vii.2018, 1♂, 1♀ and 1 nymph; **Eastern Rhodopes**: Pastrook vill., 04.iv.2012, 1♀. Detailed occurrence data: Gjonov (2022).

**Red Lists**: No assessment.

**Host plant**: Polyphagous (Emel'yanov 1967).

#### Issidae

Spinola, 1839

DE097F2C-89EA-5DF7-B04B-D98CC5394D12

#### Latilica

Emeljanov, 1971

396EA2C6-7D58-5095-888E-C8158D92D8DB

#### Latilica maculipes

(Melichar, 1906)

6241B949-ECDB-5DCF-9EBE-B1FE8F37ADC9

##### Distribution

Bosnia and Herzegovina, Croatia, Cyprus, France, Greece, Israel, Italy including the islands, Palestine, Russia (South European parts), Slovenia, Turkey, Crimea (Gnezdilov et al. 2014), Hungary (Korányi et al. 2018), Corsica (Albre and Gibernau 2019).

##### Notes

**Figure 6.**
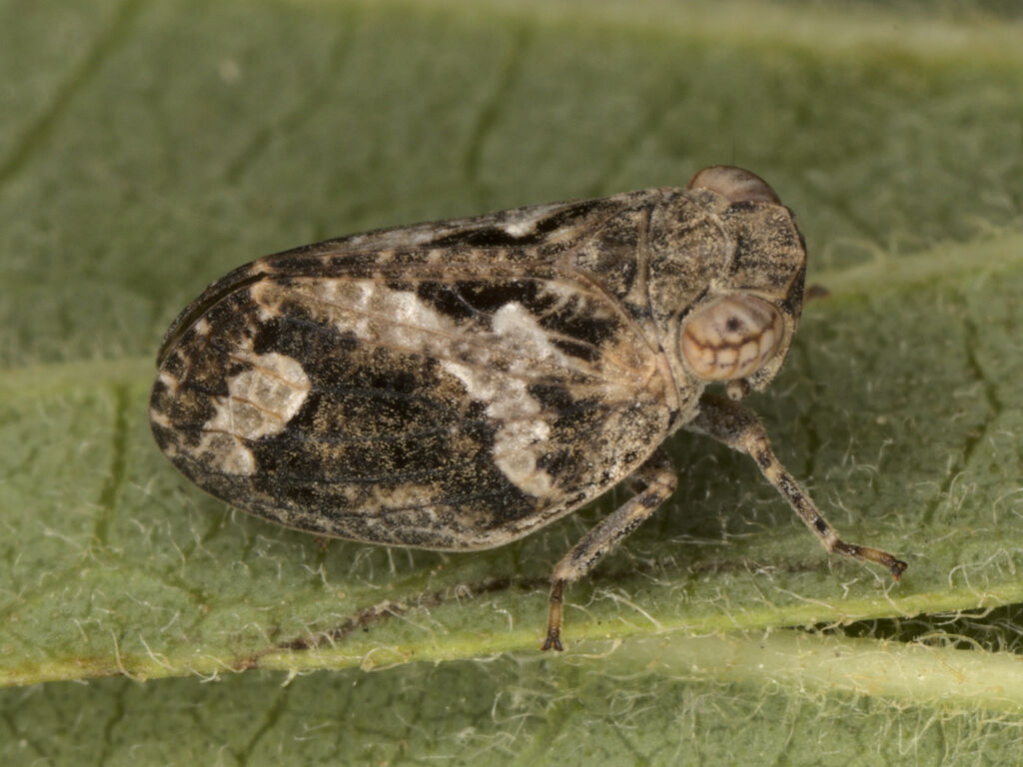
*Latilicamaculipes* (Melichar, 1906) - Bulgaria, Varna

First record for Bulgaria. **Northern Black Sea coast**: Varna, Morska gradina, 04.viii.2016, 2♂♂ and 1♀; Aksakovo vill., Pobiti kamani loc., 03.viii.2016, 5♂♂, 3♀♀ and 1 nymph; **Southern Black Sea coast**: Sinemorec vill., the mouth of Veleka River, 15.viii.2010, 1♂; Atia vill., 22.viii.2016, 1♂; **Strandzha Mt**: Pismenovo vill., 12.viii.2021, 1♂ (Fig. 6). Detailed occurrence data: Gjonov (2022).

**Red Lists**: No assessment.

**Host plant**: Polyphagous, arboreal (Korányi et al. 2018).

#### Tshurtshurnella

Kusnezov, 1927

B650C58F-B9F7-5AF8-ACA6-D5CAE97D767C

#### Tshurtshurnella extrema

Dalbola, 1980

DD63A7C6-C4BF-5747-9D83-90CA2B9EFE21

##### Distribution

Turkey, near Ankara (Dlabola 1980, Kartal 1985) and Sinop (Tanyeri and Zeybekoğlu 2021)

##### Notes

First record for Bulgaria and Europe. **Eastern Sub-Balkan Basins**: Ajtos, 01.viii.2016, 2♂♂, 2♀♀ and 3 nymphs; (Fig. 7) on or near Astracanthaarnacanthasubsp.aitosensis (Ivan.) Reer & Podlech. Detailed occurrence data: Gjonov (2022).

**Red Lists**: No assessment.

**Host plant**: Poaceae (Dlabola 1980). All specimens reported here were collected on Astracanthaarnacanthasubsp.aitosensis or near it.

## Discussion

In the current study, a list of 13 Fulgoromorpha species recorded for the first time for Bulgaria has been compiled. They are members of the families Cixiidae (one species) - *Hyalesthesmlokosiewiczi*, Delphacidae (10 species) - *Delphaxarmeniacus*, *Euidesspeciosa*, *Eurysulalurida*, *Florodelphaxparyphasma*, *Jassidaeuslugubris*, *Metropisaris*, *Oncodelphaxpullula*, *Ribautodelphaximitans*, *R.pungens*, *Stenocranusmajor* and Issidae (two species) - *Latilicamaculipes* and *Tshurtshurnellaextrema*. Additionally, the first exact localities for two species, *Tropidocephalaandropogonis* (Delphacidae) and *Dictyopharapannonica* (Dictyopharidae), are reported for Bulgaria. Species of the following five genera have not been previously known in Bulgaria: *Euides*, *Eurysula*, *Jassidaeus*, *Oncodelphax* (Delphacidae) and *Latilica* (Issidae).

As a result of the study, the total numbers of known planthopper species, genera and families in Bulgaria are now 177, 82 and 13, respectively. Although the diverse Fulgoromorpha fauna in Bulgaria has been reported so far, at least fifteen more species are expected to be discovered.

The new data significantly expand the known ranges of several species, such as *H.mlokosiewichi*, *O.pullula*, *D.armenicaus* and *T.extrema*. The easternmost distribution of *H.mlokosiewichi* (which has been found for the first time on the Balkan Peninsula) and the southernmost distribution of *O.pullula* have been established. The species *D.armenicaus*, which has been found mainly in Central Asia and the Caucasus, but is also known in Greece, is found on the Bulgarian Black Sea coast. *T.extrema* was first recorded outside of Anatolia, along with the first data on its host plant.

Seven of the listed species have conservation status in Central Europe, where such assessments have been carried out. The conservation status of most of the other species has never been evaluated as they are not spread in the countries where such assessments were carried out. This emphasises the need to assess the conservation status of Fulgoromorpha in Bulgaria.

## Supplementary Material

XML Treatment for Cixiidae

XML Treatment for Hyalesthes

XML Treatment for Hyalesthes mlokosiewiczi

XML Treatment for Delphacidae

XML Treatment for Delphax

XML Treatment for Delphax armeniacus

XML Treatment for Euides

XML Treatment for Euides speciosa

XML Treatment for Eurysula

XML Treatment for Eurysula lurida

XML Treatment for Florodelphax

XML Treatment for Florodelphax paryphasma

XML Treatment for Jassidaeus

XML Treatment for Jassidaeus lugubris

XML Treatment for Metropis

XML Treatment for Metropis aris

XML Treatment for Oncodelphax

XML Treatment for Oncodelphax pullula

XML Treatment for Ribautodelphax

XML Treatment for Ribautodelphax imitans

XML Treatment for Ribautodelphax pungens

XML Treatment for Stenocranus

XML Treatment for Stenocranus major

XML Treatment for Tropidocephala

XML Treatment for Tropidocephala andropogonis

XML Treatment for Dictyopharidae

XML Treatment for Dictyophara

XML Treatment for Dictyophara pannonica

XML Treatment for Issidae

XML Treatment for Latilica

XML Treatment for Latilica maculipes

XML Treatment for Tshurtshurnella

XML Treatment for Tshurtshurnella extrema

## Figures and Tables

**Figure 1a.**
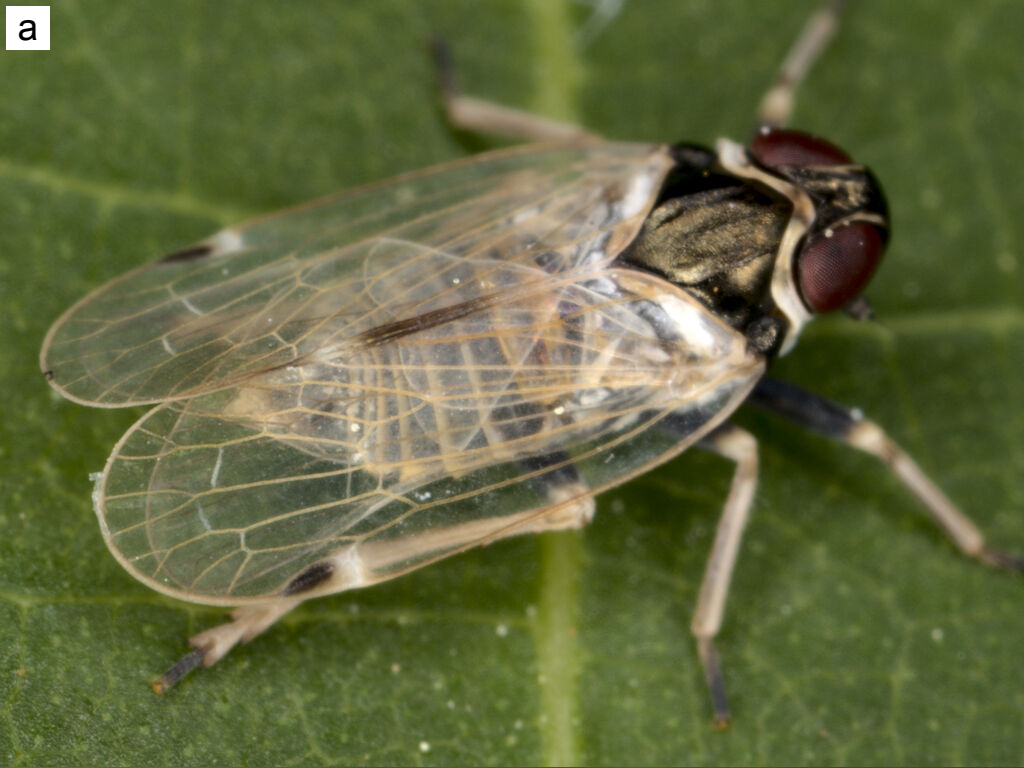
dorsal view

**Figure 1b.**
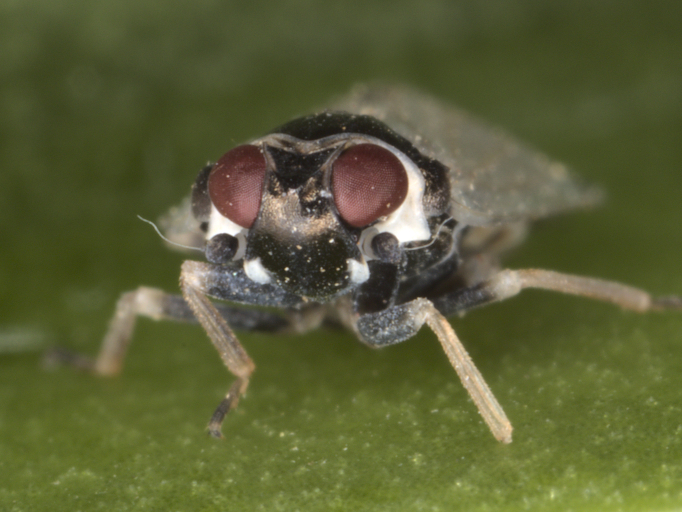
frontal view

**Figure 2a.**
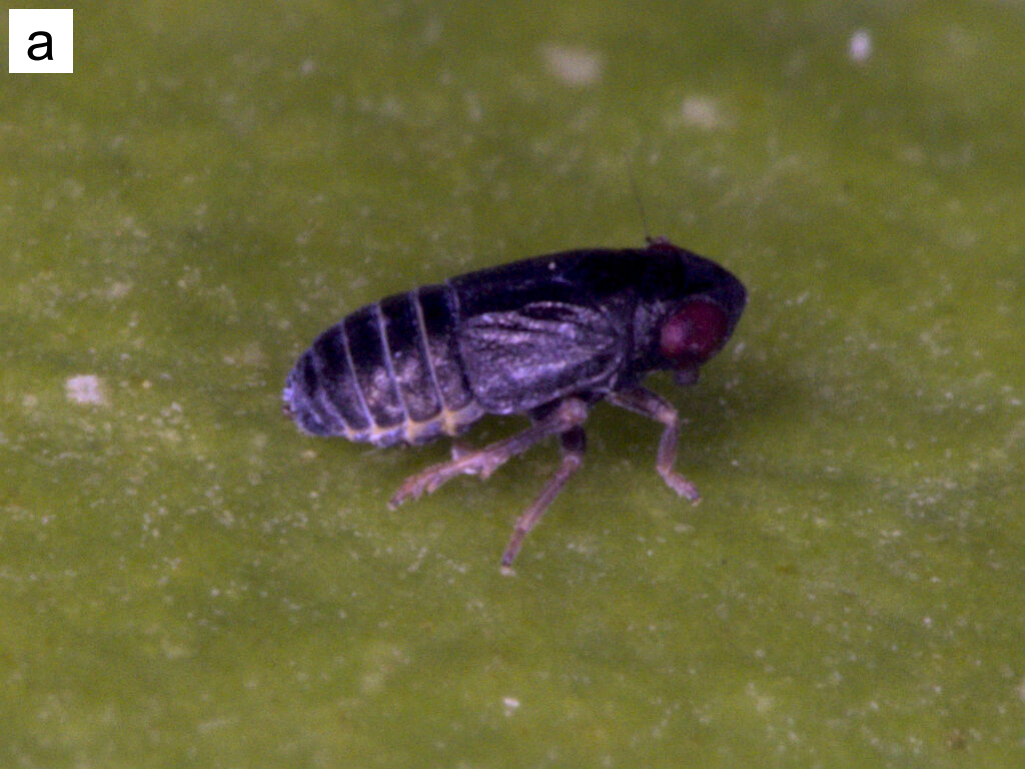
male

**Figure 2b.**
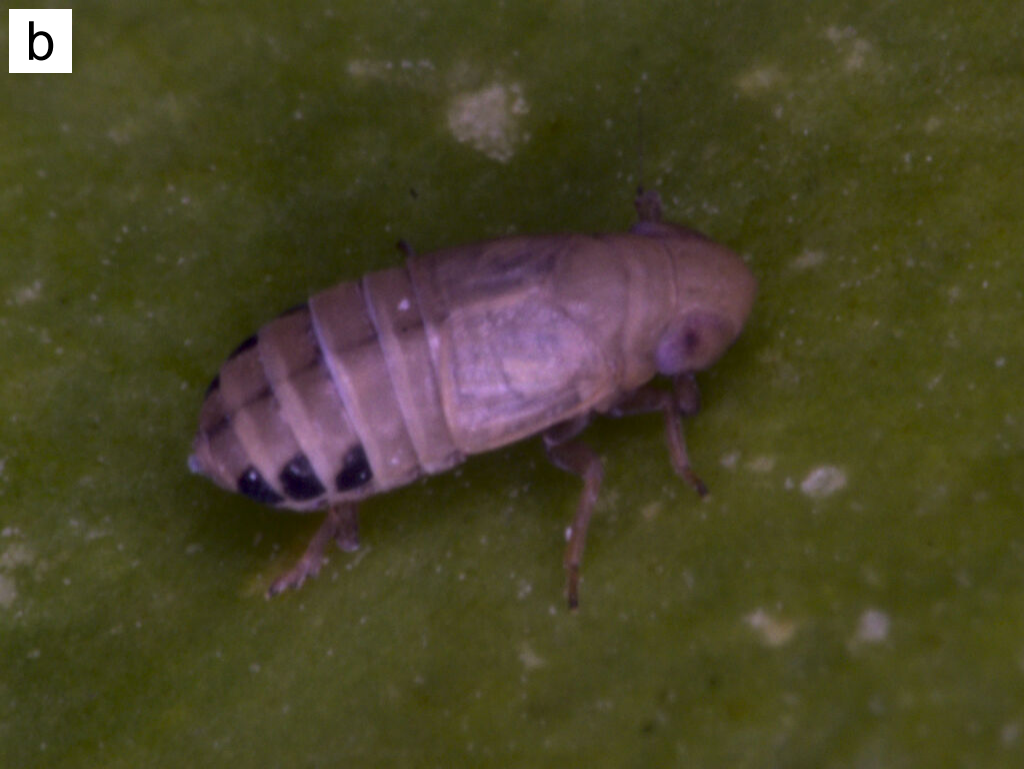
female

**Figure 4a.**
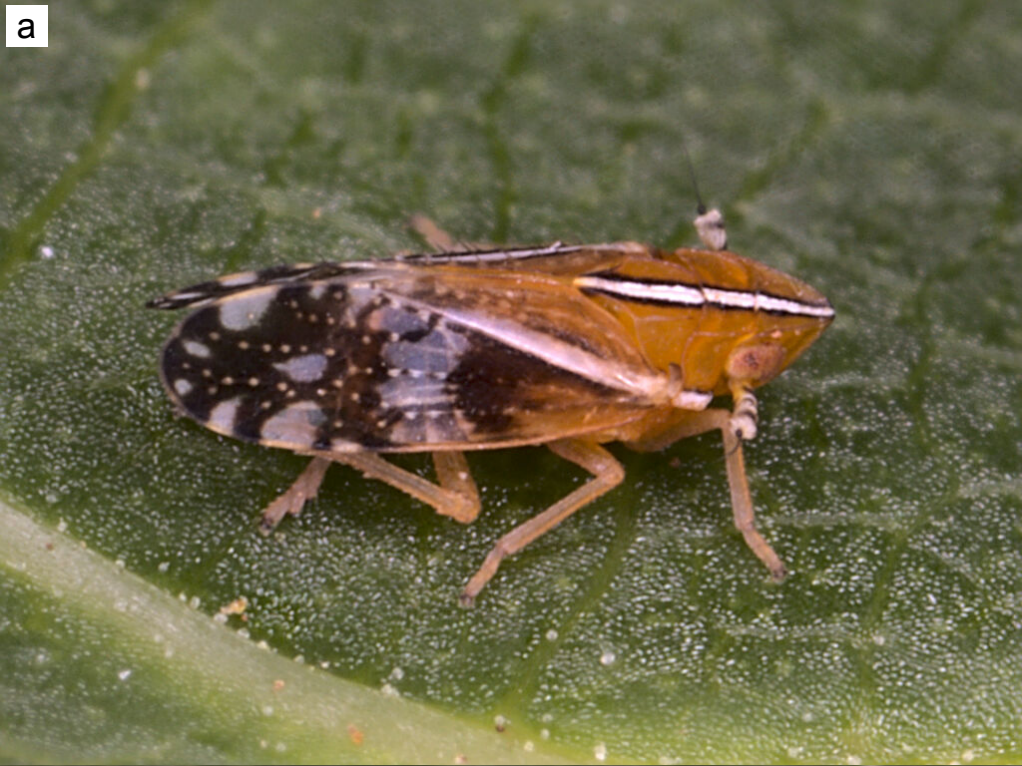
male

**Figure 4b.**
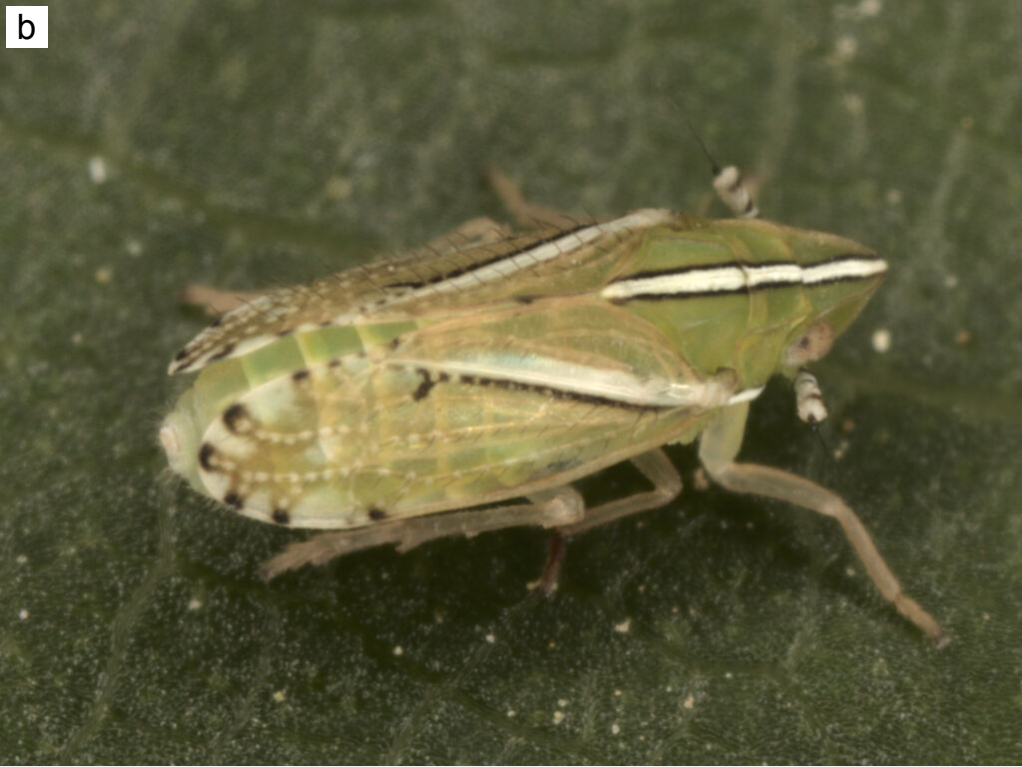
female

**Figure 5a.**
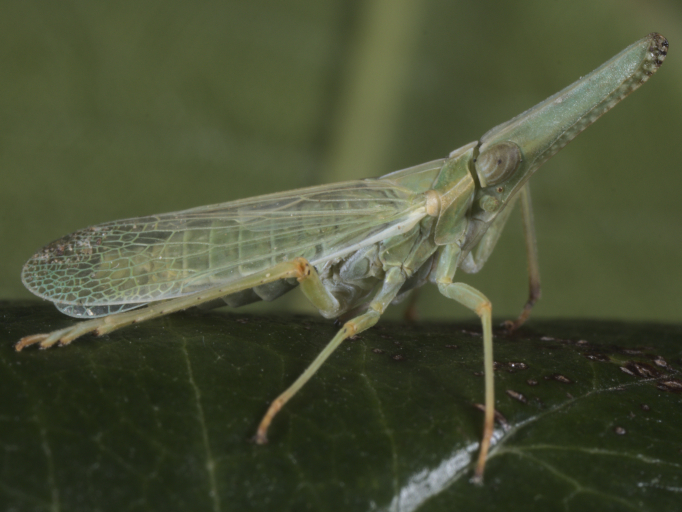
lateral view

**Figure 5b.**
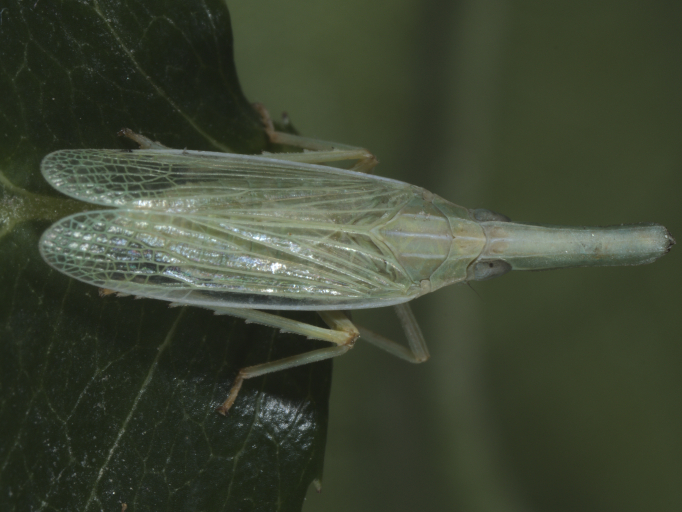
dorsal view

**Figure 7a.**
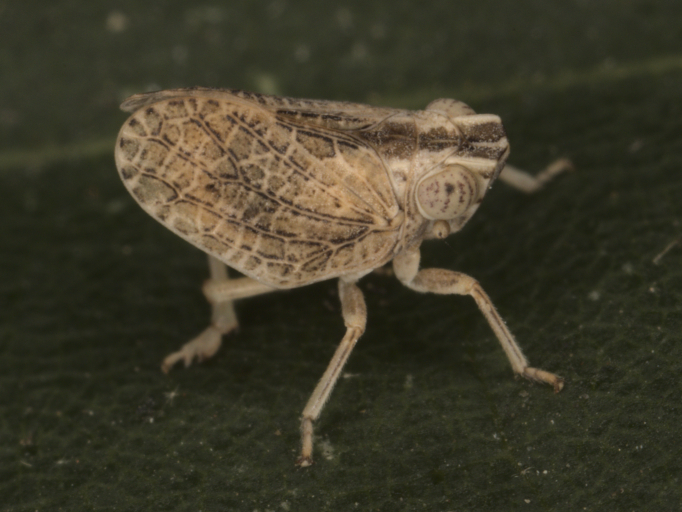
lateral view

**Figure 7b.**
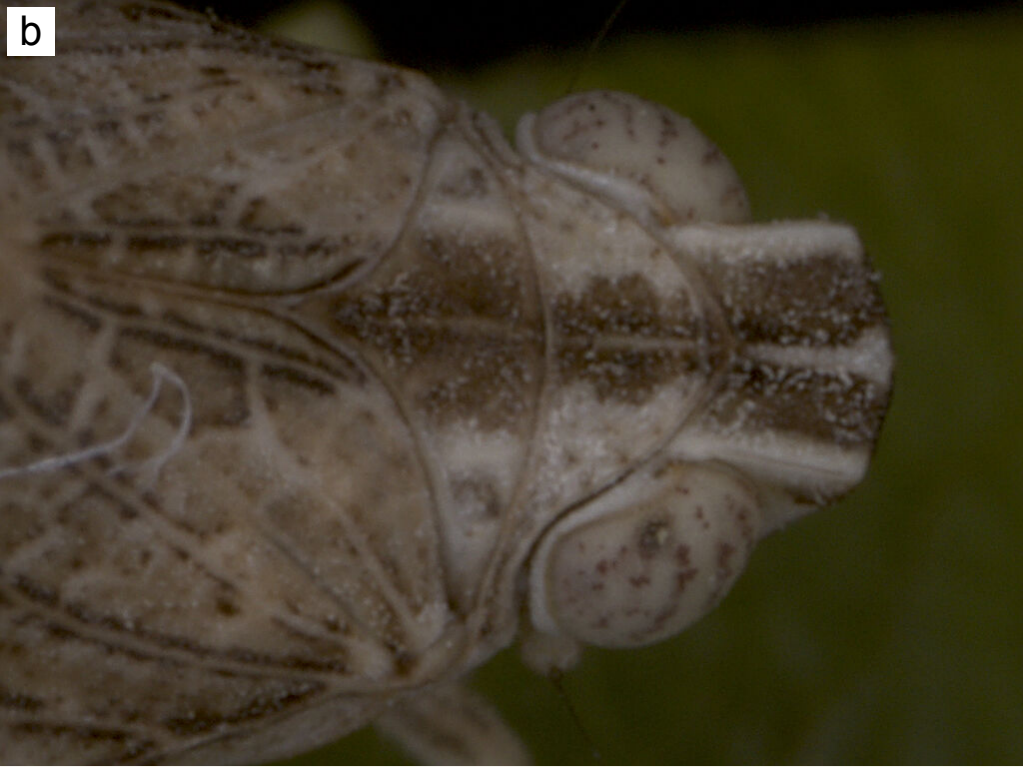
dorsal view of the head

## References

[B7712820] Aguin-Pombo Dora, Freitas Carlos, Alvaréz Pedro, Bourgoin Thierry (2007). Catálogo de los hemípteros, Cicadomorpha y Fulgoromorpha de Aragón. Catalogus de la Entomofauna Aragonesa.

[B7716451] Aguin-Pombo Dora, Freitas Carlos, Alvaréz Pedro A., Bourgoin Thierry (2008). A bibliographic catalogue of the Cicadomorpha and Fulgoromorpha of North East Spain (Aragon). Bulletin of Insectology.

[B7826400] Albre Jérôme, Gibernau Marc (2019). Diversity and temporal variations of the Hemiptera
Auchenorrhyncha fauna in the Ajaccio region (France, Corsica). Annales de la Société entomologique de France (N.S.).

[B7834372] Anufriev G. A. (1970). Two new Palaearctic species of *Delphax* Fabricius, 1798 (Homoptera, Delphacidae). Bulletin de l’Académie Polonaise des Sciences. Série des Sciences Biologiques.

[B7712905] Anufriev Georgy Aleksandrovich (2002). New and little known species of cycadoids of the family Delphacidae (Homoptera, Cicadinea) from North Kyrgyzstan. Entomological Investigatons in Kirgyzstan.

[B7716700] Anufriev G A, Bayanov N G (2002). [Invertebrates fauna of Kerzhensky Reserve according to studies in 1993–2001. Materials on fauna of Nizhny Novgorod Trans-Volga region. Nizhny Novgorod]. [Proceedings of the State Natural Biosphere Reserve «Kerzhensky»].

[B7712545] Asche Manfred (1982). Beiträge zur Delphaciden-Fauna Ungarns (Homoptera
Cicadina
Delphacidae). Marburger Entomologische Publikationen.

[B7712757] Asche Manfred (1982). Beiträge zur Delphaciden - Fauna Jugoslawiens und Bulgariens (Homoptera
Cicadina
Delphacidae). Marburger Entomologische Publikationen.

[B7713035] Bartlett Charles R. (2009). Diversity in New World Stenocraninae Planthoppers (Hemiptera: Delphacidae). Transactions of the American Entomological Society.

[B7716460] Baugnée Jean-Yves (2004). Contribution a la connaissance des Delphacidae de Belgique (Hemiptera
Auchenorrhyncha
Fulgoromorpha). Bulletin de la Société royale belge d'Entomologie.

[B7713070] Biedermann Robert, Niedringhaus Rolf (2009). The Plant- and Leafhoppers of Germany - Identification Key to all Species.

[B7716469] Bonfils Jacques, Della Giustina William (1978). Inventaire et répartition biogéographique des Homoptères Auchénorhynques de Corse. Bulletin de la Société entomologique de France.

[B7712793] Borodin Oleg (2004). A checklist of the Auchenorrhyncha of Belarus (Hemiptera, Fulgoromorpha et Cicadomorpha). Beiträge zur Zikadenkunde.

[B7727590] Bourgoin Thierry FLOW (Fulgoromorpha Lists on The Web): a world knowledge base dedicated to Fulgoromorpha. Version 8. https://flow.hemiptera-databases.org/flow/.

[B7723821] Bückle Christoph, Guglielmino Adalgisa (2011). Zur Zikadenfauna (Auchenorrhyncha) im Umland von Fließgewäs- sern und Quellen am Hohen Trauchberg, Ostallgäu/Bayerische Alpen. Lauterbornia.

[B7712775] Carl Michael (2008). Die Zikaden (Insecta, Auchenorrhyncha) des Schlern (Südtirol, Italien). Gredleriana.

[B7712426] Cvrković Tatjana, Mitrović Milana, Jović Jelena, Krnjajić Slobodan, Krstić Oliver, Toševski Ivo (2010). Diversity of cicads (Hemiptera: Auchenorrhyncha) in Serbian vineyards. Zaštita bilja.

[B7712454] Cvrković Tatjana, Jović Jelena, Mitrović Milana, Krstić Oliver, Krnjajić Slobodan, Toševski Ivo (2011). Potential new hemipteran vectors of stolbur phytoplasma in Serbian vineyards. Bulletin of Insectology.

[B7716478] Della Giustina William, Remane Reinhard (2001). Compléments à la faune de France des Auchenorrhyncha: espèces et données additionnelles; modifications à l'ouvrage de Nast (1987) (Homoptera). Bulletin de la Société entomologique de France.

[B7716435] Della Giustina William (2019). Les Delphacidae de France et des pays limitrophes (Hemiptera, Fulgoromorpha) Tome 1&2.

[B7713163] Demir Emine (2007). Contributions to the knowledge of Turkish Auchenorrhyncha (Homoptera, Fulgoromorpha and Cicadomorpha, excl. Cicadellidae) with a new record, *Setapiusklapperichianus* Dlabola, 1988. Munis Entomology & Zoology.

[B7713154] Demir Emine, Demirsoy Ali (2009). Preliminary report on the Fulgoromorpha (Hemiptera) fauna of Kemaliye (Erzincan) with a new record for Turkey. Munis Entomology & Zoology.

[B7826382] Demirel Ersin, Hasbenli Abdullah (2015). Contributions to the Bolkar Mountains Cixiidae fauna with a new record and an identification key for Turkey's *Tachycixius* (Hemiptera: Auchenorrhyncha). Pakistan Journal of Zoology.

[B7712740] den Bieman C F M (1987). Biological and taxonomie differentiation in the *Ribautodelphaxcollinus* complex (Homoptera, Delphacidae).

[B7712968] den Bieman C F M (1988). Coexistence of pseudogamous and sexual planthoppers of the genus *Ribautodelphax* (Homoptera, Delphacidae). Ecological Entomology.

[B7826439] den Bieman C F M (1993). Het spoorcicaden genus *Stenocranus* in Nederland (Homoptera: Delphacidae). Entomologische Berichten.

[B7826430] den Bieman C F M, Mol A W M (2010). Vier soorten spoorcicaden voor het eerst in Nederland aangetroffen (Hemiptera, Fulgoromorpha, Delphacidae). Entomologische Berichten.

[B7826448] den Bieman C F M, Tansi Marco, Drukker Eva F, de Waart Sytske (2021). The leafhopper fauna of green roofs including the Mediterranean leaf- hopper *Circuliferhaematoceps* new for the Netherlands (Auchenorrhyncha: Cicadellidae). Entomologische berichten.

[B7716487] Dlabola Jiří (1950). Homopterologické zajímavosti Slovenska. Some records of leafhoppers from Slovakia. (Homoptera, Auchenorrhyncha). Acta Societatis entomologicae Čechosloveniae.

[B7826413] Dlabola Jiří (1954). Ergänzungen zur Faunistik der ČSR und Ungarns mit der Beschreibung einer neuen *Typhlocyba*-Art (Hom., Auchenorrhyncha. Acta Societatis entomologicae Čechosloveniae.

[B7712851] Dlabola Jiří (1957). Results of the zoological expedition of the National Museum in Prague to Turkey. 20. Homoptera
Auchenorrhyncha. Acta Entomologica Musei Nationalis Pragae.

[B7712687] Dlabola Jiří (1958). Zikaden-Ausbeute vom Kaukasus (Homoptera
Auchenorrhyncha). Acta Entomologica Musei Nationalis Pragae.

[B7713087] Dlabola Jiří (1959). Fünf neue Zikaden-Arten aus dem Gebiet des Mittelmeers. Bollettino della Societa Entomologica Italiana.

[B7712860] Dlabola Jiří (1977). Homoptera
Auchenorrhyncha. In: Enumeratio Insectorum bohemoslovakiae. Check List Tschechoslowakische Insektenfauna. Acta Faunistica Entomologica Musei Nationalis Pragae.

[B7723741] Dlabola Jiří (1980). Tribus-Einteilung, neue Gattungen und Arten der Subf. Issinae in der eremischen Zone (Homoptera, Auchenorrhyncha). Sborník Národního Muzea v Praze, Řada B, Přírodní Vědy.

[B7712731] Dlabola Jiří (1981). Ergebnisse der tschechoslowakisch-iranischen entomologischen Expeditionen nach dem Iran (1970 und 1973). (Mit Angaben über einiger Sammelresultate in Anatolien). Homoptera: Auchenorrhyncha (II. Teil). Acta Entomologica Musei Nationalis Pragae.

[B7712722] Drosopoulos Sakis (1982). *Remanodelphaxcedroni* gen. et spec. nov. from Greece (Homoptera, Auchenorrhyncha, Delphacidae). Marburger Entomologische Publikationen.

[B7713061] Drosopoulos Sakis (1982). Hemipterological Studies in Greece. Part II. Homoptera - Auchenorrhyncha. On the Family Delphacidae.. Marburger Entomologische Publikationen.

[B7712678] Drosopoulos Sakis, Asche Manfred, Hoch Hannelore (1983). Contribution to the planthopper fauna of Greece. Homoptera, Auchenorrhyncha, Fulgoromorpha, Delphacidae). Annales de l'Institut Phytopathologique Benaki (N.S.).

[B7712665] D'Urso Vera, Minelli A, Ruffo S, La Porta S (1995). Checklist delle specie della fauna italiana.

[B7712483] Emeljanov Alexandr Fedorovich (2015). Planthoppers of the family Cixiidae of Russia and adjacent territories. Key to the fauna of Russia.

[B7716496] Emel'yanov Alexandr Fedorovich, Bei-Bienko G. Ya. (1967). Keys to the Insects of the European USSR. Vol.1.

[B7712869] Gębicki Cezary, Świerczewski Dariusz, Szwedo Jacek (2013). Planthoppers and leafhoppers of Poland (Hemiptera: Fulgoromorpha et Cicadomorpha) Systematics. Check-list. Bionomy. Annals of the Upper Silesian Museum in Bytom. Entomology.

[B7727581] Gjonov I. (2022). new_fulgoromorpha_records_bulgaria_2022. Biodiversity Data Journal. Occurrence dataset.

[B7716517] Gnezdilov V. M., Holzinger W. E., Wilson Michael R. (2014). The Western Palaearctic Issidae (Hemiptera, Fulgoroidea): an Illustrated Checklist and Key to Genera and Subgenera. Proceedings of the Zoological Institute RAS.

[B7712474] Gravestein W H (1976). Naamlijst van de in Nederland voorkomende Cicaden (Homoptera, Auchenorrhyncha). Entomologische Berichten.

[B7712784] Guglielmino Adalgisa, Bückle Christoph, Remane Reinhard (2005). Contribution to the knowledge of the Auchenorrhyncha fauna of Central Italy (Hemiptera, Fulgoromorpha et Cicadomorpha). Marburger Entomologische Publikationen.

[B7712630] Guglielmino Adalgisa, Bückle Christoph (2008). Contribution to the knowledge on the Auchenorrhyncha fauna (Hemiptera
Fulgoromorpha et Cicadomorpha) of the Tuscanian-Emilian Apennines. Redia.

[B7712621] Guglielmino Adalgisa, Olmi Massimo, Bückle Christoph (2013). An updated host-parasite catalogue of world Dryinidae (Hymenoptera: Chrysidoidea). Zootaxa.

[B7712923] Györffy György, Kiss Balázs, Koczor Sándor, Orosz András (2009). Checklist of the fauna of Hungary. Volume 4. Hemiptera: Archaeorrhyncha, Clypeorrhyncha.

[B7716526] Hayashi M., Fujinuma Satoshi, Japan Editorial Committee of Catalogue of the Insects of (2016). Catalogue of the Insects of Japan Volume 4 Paraneoptera (Psocodea, Thysanoptera, Hemiptera).

[B7713219] Hoch Hannelore (1990). New synonyms and records in the cixiid genus *Hyalesthes* Signoret, 1865 (Hom., Fulgoroidea). Entomologist's Monthly Magazine.

[B7727598] Hoch Hannelore Fauna Europaea: Fulgoromorpha. http://www.faunaeur.org.

[B7712594] Holzinger Werner E. (1996). Die Zikadenfauna wärmeliebender Eichenwälder Ostösterreichs (Insecta: Homoptera, Auchenorrhyncha). Mitteilungen des Naturwissenschaftlichen Vereins für Steiermark.

[B7712656] Holzinger Werner E. (1996). Kritisches Verzeichnis der Zikaden Österreichs (Ins.: Homoptera, Auchenorrhyncha). Carinthia II.

[B7712647] Holzinger Werner E., Seljak Gabrijel (2001). New Records of Planthoppers and Leafhoppers from Slovenia, with a checklist of hitherto recorded species (Hemiptera: Auchenorrhyncha). Acta Entomologica Slovenica.

[B7712639] Holzinger Werner E., Kammerlander Ingrid, Nickel Herbert (2003). The Auchenorrhyncha of Central Europe – Die Zikaden Mitteleuropas Volume 1: Fulgoromoropha, Cicadomorpha excl. Cicadellidae.

[B7712518] Holzinger Werner E., Kunz Gernot (2006). New records of leafhoppers and planthoppers from Austria (Hemiptera: Auchenorrhyncha). Acta Entomologica Slovenica.

[B7727606] Holzinger W. E. (1999). Rote Liste der Zikaden Kärntens (Insecta: Auchenorrhyncha). Naturschutz in Kärnten.

[B7834552] Holzinger W. E., Zulka Klaus Peter (2009). Rote Listen gefährdeter Tiere Österreichs. Checklisten, Gefährdungsanalysen, Handlungsbedarf.

[B7716539] Holzinger W E, Aukema B, den Bieman C F M, Bourgoin T, Burck-hardt D, Carapezza A, Cianferoni F, Chen P P, Faraci F, Goula M (2017). Hemiptera records from Lake Spechtensee and from Southern Styria (Austria). Entomologica Austriaca.

[B7826391] Holzinger Werner E, Huber Elisabeth, Schlosser Lydia, Kunz Gernot (2020). *Acanaloniaconica* (Say, 1830) and three other true hopper species new for Austria (Hemiptera: Auchenorrhyncha). Cicadina.

[B7712931] Horváth Géza (1895). Hemipteres nouveaux d'Europe et des pays limitrophes. Revue d'Entomologie.

[B7716563] Kahapka Jördis, Kunz Gernot, Nationalpark Gesäuse GmbH Weng im Gesäuse (2011). Schriften des Nationalparks Gesäuse.

[B7727564] Kartal V. (1985). Türkiye’den az bilinen *Tshurtshurnellaextrema* Dlabola, 1980 (Homoptera, Auchenorrhyncha, Issidae) türü. Doga Bilim Dergisi.

[B7834428] Korányi Dávid, Markó Viktor, Haltrich Attila, Orosz András (2018). First records of *Latilicamaculipes* (Hemiptera: Issidae) and *Synophropsislauri* (Hemiptera: Cicadellidae) in Hungary. Opuscula Zoologica Budapest.

[B7712940] Kunz Gernot, Plank C. (2002). Zikaden im Nationalpark Gesäuse unter Berücksichtigung aktueller Aufsammlungen (Hemiptera: Auchenorrhyncha). Entomologica Austriaca.

[B7712509] Kunz Gernot (2010). Erste Zikadenerhebungen im Nationalpark Thayatal (Insecta, Auchenorrhyncha). Wissenschaftliche Mitteilungen aus dem Niederösterreichischen Landesmuseum.

[B7716576] Kunz Gernot, Kahapka Jördis (2012). Zikaden (Insecta: Hemiptera: Auchenorrhyncha) im Kalktal bei Hieflau. Abhandlungen der Zoologisch-botanischen Gesellschaft in Österreich.

[B7712995] Le Quesne Walter J., Payne K. R. (1981). Cicadellidae (Typhlocybinae) with a check list of the British Auchenorhyncha (Hemiptera, Homoptera). Handbooks for the identification of British insects.

[B7712949] Lessio Federico, Alma Alberto (2008). Host plants and seasonal presence of *Dictyopharaeuropaea* in the vineyard agro-ecosystem. Bulletin of Insectology.

[B7713181] Lindberg P. Håkan (1960). Über Zikaden von Sowjetarmenien. Notulae entomologicae.

[B7826373] Lodos Niyazi, Kalkandelen Ayla (1980). Preliminary list of Auchenorrhyncha with notes on distribution and importance of species in Turkey I. Family Cixiidae Spinola. Türkiye Bitki Koruma Dergisi.

[B7713017] Logvinenko V N (1975). Fulgoroidny cicadovy Fulgoroidea. Fauna Ukrainy.

[B7713105] Malenovský Igor (2006). Planthoppers and leafhoppers (Auchenorrhyncha, Hemiptera) of Kokorínsko Protected Landscape Area. Bohemia Centralis.

[B7713114] Malenovský Igor, Lauterer Pavel (2010). Additions to the fauna of planthoppers and leafhoppers (Hemiptera: Auchenorrhyncha) of the Czech Republic. Acta Musei Moraviae, Scientiae Biologicae.

[B7712612] Malenovský Igor, Baňař Petr, Kment Petr (2011). A contribution to the faunistics of the Hemiptera (Cicadomorpha, Fulgoromorpha, Heteroptera, and Psylloidea) associated with dry grassland sites in southern Moravia (Czech Republic). Acta Musei Moraviae, Scientiae Biologicae.

[B7712603] Malenovský Igor, Lauterer Pavel (2012). Leafhoppers and planthoppers (Hemiptera: Auchenorrhyncha) of the Bílé Karpaty Protected Landscape Area and Biosphere Reserve (Czech Republic). Acta Musei Moraviae, Scientiae Biologicae.

[B7712536] Malenovský Igor, Kment Petr, Sychra Jan (2014). Faunistics, insects, Cicadomorpha, Fulgoromorpha, Heteroptera, Sternorrhyncha, Erzgebirge, Bohemia, central Europe, peat bogs, tyrphobionts, tyrphophilous fauna. Klapalekiana.

[B7713123] Malenovský Igor, Lauterer Pavel, Farkač Jan, Král David, Škorpík Martin (2017). Red list of threatened species in the Czech Republic. Invertebrates.

[B7712527] Melichar Leopold (1914). Zweiter Beitrag zur Kenntnis der kaukasischen Homopterenfauna. Mitteilungen des Kaukasischen Museums.

[B7716594] Metcalf Zeno P (1943). General Catalogue of the Hemiptera, Fascicle IV, Fulgoroidea, Part 3, Araeopidae (Delphacidae).

[B7716602] Mitjaev I D (1971). Leafhoppers of Kazakhstan (Homoptera-Cicadinea). Science of Kazakh SSR.

[B7834889] Mitjaev I. D. (2015). Leafhoppers (Homoptera, Cicadinea) of Kazakhstan, annotated check-list of species. Selevinia.

[B7712959] Mozaffarian Fariba, Wilson Michael R. (2011). An annotated checklist of the planthoppers of Iran (Hemiptera, Auchenorrhyncha, Fulgoromorpha) with distribution data.. ZooKeys.

[B7713239] Mozaffarian Fariba (2014). Fauna of planthoppers superfamily Fulgoroidea (Hem.: Auchenorrhyncha) in the northwestern Iran. Journal of Field Crop Entomology.

[B7712977] Mozaffarian Fariba (2018). An Identification key to the species of Auchenorrhyncha of Iranian fauna recorded as pests in orchards and a review on the pest status of the species. Zootaxa.

[B7834856] Mühlethaler Roland, Holzinger Werner E, Nickel Herbert, Wachmann Ekkehard, Meyer Quelle & (2018). Die Zikaden Deutschlands, Österreichs und der Schweiz: Entdecken – Beobachten – Bestimmen.

[B7716620] Nast Janusz (1972). Palaearctic Auchenorrhyncha (Homoptera). An annotated check list.

[B7727628] Nast J. (1986). Notes on some Auchenorrhyncha (Homoptera) VI - X. Annales Zoologici.

[B7716611] Nast Janusz (1987). The Auchenorrhyncha (Homoptera) of Europe. Annales zoologici.

[B7713096] Nickel Herbert, Remane Reinhard (2002). Check list of the planthoppers and leafhoppers of Germany, with notes on food plants, diet width, life cycles, geographic range and conservation status (Hemiptera, Fulgoromorpha and Cicadomorpha). Beiträge zur Zikadenkunde.

[B7716628] Nickel Herbert (2003). Rote Liste gefährdeter Zikaden (Hemiptera, Auchenorrhyncha) Bayerns. Schriftenreihe des Bayerischen Landesamtes für Umweltschutz.

[B7833465] Nickel H. (2003). The Leafhoppers and Planthoppers of Germany (Homoptera, Auchenorrhyncha): Patterns and strategies in a highly diverse group of phytophagous insects.

[B7712766] Nickel Herbert, Remane Reinhard (2003). Verzeichnis der Zikaden (Auchenorrhyncha) der Bundesländer Deutschlands. Entomofauna Germanica.

[B7723812] Nickel Herbert, Gärtner Eberhard (2009). Tyrphobionte und tyrphophile Zikaden (Hemiptera, Auchenorrhyncha) in der Hannoverschen Moorgeest – Biotopspezifische Insekten als Zeigerarten für den Zustand von Hochmooren. TELMA.

[B7712811] Nickel Herbert, Sander Friedrich Wilhelm (2016). Rote Liste der Zikaden (Insecta: Hemiptera: Auchenorrhyncha) Thüringens. Landschaftspflege und Naturschutz in Thüringen.

[B7716637] Nickel Herbert, Achtziger Roland, Biedermann Robert, Bückle Christoph, Deutschmann U, Niedringhaus Rolf, Remane Reinhard, Walter S., Witsack Werner (2016). Rote Liste und Gesamtartenliste der Zikaden. Naturschutz und Biologische Vielfalt.

[B7712748] Niedringhaus Rolf, Biedermann Robert, Nickel Herbert (2010). Verbreitungsatlas der Zikaden des Großherzogtums Luxemburg - Textband. Ferrantia.

[B7712914] Niedringhaus Rolf, Biedermann Robert, Nickel Herbert (2010). Verbreitungsatlas der Zikaden des Großherzogtums Luxemburg - Atlasband. Ferrantia.

[B7712572] Orosz András (2008). Contributions to the leafhopper fauna of the protected areas along the river Tur (Homoptera: Auchenorrhyncha). Biharean Biologist.

[B7712696] Orosz András (2009). Gyűrűfűn a Biodiverzitás Napokon gyűjtött kabócák (Auchenorrhyncha). Natura Somogyensis.

[B7716651] Orosz András, Tóth Mária (2016). Contribution to the Auchenorrhyncha fauna of Şalaj county, Romania. Studia Universitatis “Vasile Goldiş”, Seria Ştiinţele Vieţii.

[B7712437] Park Jaekook, Jung Sunghoon (2020). Two newly recorded genera and species of the plant hopper (Hemiptera: Auchenorhyncha: Delphacidae) in Korea. Journal of Asia-Pacific Biodiversity.

[B7716660] Popa Valentin, Popa Alina (2002). New records of the Auchenorrhyncha (Hemiptera) species in the fauna of Romania. Acta Entomologica Slovenica.

[B7713052] Preisler Jiří, Lauterer Pavel (2003). Some new species of planthoppers and leafhoppers for the Czech Republic and Slovakia. Beiträge zur Zikadenkunde.

[B7716669] Remane Reinhard, Fröhlich Wolfgang (1994). Beiträge zur Chorologie einiger Zikaden- Arten (Homoptera
Auchenorrhyncha) in der Westpaläarktis. Marburger Entomologische Publikationen.

[B7713145] Seljak Gabrijel (2004). Contribution to the knowledge of planthoppers and leafhoppers of Slovenia (Hemiptera: Auchenorrhyncha). Acta Entomologica Slovenica.

[B7713136] Seljak Gabrijel (2016). New and little known plant- and leafhoppers of the fauna of Slovenia (Hemiptera: Fulgoromorpha and Cicadomorpha). Acta Entomologica Slovenica.

[B7713172] Smirnova N V, Anufriev G A (2014). [On cicadina fauna (Homoptera, Cicadina) of «Kerzhensky» Reserve]. [Proceedings of the State Natural Biosphere Reserve «Kerzhensky»].

[B7712829] Söderman Guy, Gillerfors Gosta, Endrestøl Anders, Söderman Guy (2009). An annotated catalogue of the Auchenorrhyncha of Northern Europe. Cicadina.

[B7712465] Song Zhi Shun, Liang A. I.Piing (2008). The palaearctic planthopper genus *Dictyophara* Germar, 1833 (Hemiptera: Fulgoroidea: Dictyopharidae) in China. Annales Zoologici.

[B7723732] Tanyeri Rukiye, Zeybekoğlu Ünal (2021). Species of Cixiidae and Issidae (Hemiptera: Auchenorrhyncha: Fulgoromorpha) distributed in Sinop and Kastamonu (Turkey). Sakarya University Journal of Science.

[B7712986] Walczak Marcin (2016). The fauna of planthoppers and leafhoppers (Hemiptera: Fulgoromorpha et Cicadomorpha) in the city of Częstochowa (southern Poland). Annals of the Upper Silesian Museum in Bytom. Entomology.

[B7712581] Walter Sabine, Emmrich Rainer, Nickel Herbert, Landschaftsschutz Abt. Natur- und (2003). Materialien zu Naturschutz und Landschaftspflege.

[B7713026] Witsack Werner, Nickel Herbert (2004). Rote Liste der Zikaden (Hemiptera, Auchenorrhyncha) des Landes Sachsen-Anhalt. Berichte des Landesamtes für Umweltschutz Sachsen-Anhalt.

